# Analysis of Content Shared in Online Cancer Communities: Systematic Review

**DOI:** 10.2196/cancer.7926

**Published:** 2018-04-03

**Authors:** Mies C van Eenbergen, Lonneke V van de Poll-Franse, Emiel Krahmer, Suzan Verberne, Floortje Mols

**Affiliations:** ^1^ Department of Research Netherlands Comprehensive Cancer Organisation Utrecht Netherlands; ^2^ Division of Psychosocial Research & Epidemiology The Netherlands Cancer Institute Amsterdam Netherlands; ^3^ Department of Medical and Clinical Psychology Tilburg University Tilburg Netherlands; ^4^ Tilburg Center for Cognition and Communication Tilburg University Tilburg Netherlands; ^5^ Leiden Institute of Advanced Computer Science Leiden University Leiden Netherlands

**Keywords:** cancer, survivors, support groups, internet

## Abstract

**Background:**

The content that cancer patients and their relatives (ie, posters) share in online cancer communities has been researched in various ways. In the past decade, researchers have used automated analysis methods in addition to manual coding methods. Patients, providers, researchers, and health care professionals can learn from experienced patients, provided that their experience is findable.

**Objective:**

The aim of this study was to systematically review all relevant literature that analyzes user-generated content shared within online cancer communities. We reviewed the quality of available research and the kind of content that posters share with each other on the internet.

**Methods:**

A computerized literature search was performed via PubMed (MEDLINE), PsycINFO (5 and 4 stars), Cochrane Central Register of Controlled Trials, and ScienceDirect. The last search was conducted in July 2017. Papers were selected if they included the following terms: (cancer patient) and (support group or health communities) and (online or internet). We selected 27 papers and then subjected them to a 14-item quality checklist independently scored by 2 investigators.

**Results:**

The methodological quality of the selected studies varied: 16 were of high quality and 11 were of adequate quality. Of those 27 studies, 15 were manually coded, 7 automated, and 5 used a combination of methods. The best results can be seen in the papers that combined both analytical methods. The number of analyzed posts ranged from 200 to 1,500,000; the number of analyzed posters ranged from 75 to 90,000. The studies analyzing large numbers of posts mainly related to breast cancer, whereas those analyzing small numbers were related to other types of cancers. A total of 12 studies involved some or entirely automatic analysis of the user-generated content. All the authors referred to two main content categories: informational support and emotional support. In all, 15 studies reported only on the content, 6 studies explicitly reported on content and social aspects, and 6 studies focused on emotional changes.

**Conclusions:**

In the future, increasing amounts of user-generated content will become available on the internet. The results of content analysis, especially of the larger studies, give detailed insights into patients’ concerns and worries, which can then be used to improve cancer care. To make the results of such analyses as usable as possible, automatic content analysis methods will need to be improved through interdisciplinary collaboration.

## Introduction

### Background

In recent years, the concept of *online community* for patients and their relatives (ie, posters) has developed as a result of improved technical possibilities [1]. Literature cites various forms of online contact between patients, including bulletin boards, closed networks, mailing lists, newsgroups, discussion forums (moderated or otherwise), chat rooms, Facebook groups, Twitter follow groups, email groups, etc. [2-4]. Furthermore, patients—as well as their family members and friends—have come to relate to these environments, partly because of the popularity of Facebook and other social platforms [5]. Sharing experiences may help patients to understand their illness and compare their situation. They possibly learn from others [6], have more access to services, and support better (shared) decisions about health care, such as treatment options [7,8].

Nowadays, there are an increasing number of online health communities, for cancer and other diseases, each with its own specific aims. As a potentially life-threatening illness with a growing number of new patients and survivors [9], cancer can raise a wide range of specific informational and emotional support issues [10]. Also, patients have much experiential knowledge that can be relevant to others. They share such knowledge also in online communities. Through interaction with each other, they not only share experiences and raise awareness for certain issues among themselves but also among health care providers and the research community [11].

Research into (the effects on individuals) participating in online communities can roughly be divided into 2 main variants: first, researchers can ask community participants to complete one or more questionnaires, thereby measuring the effects of participation on the individual; second, researchers can analyze content that has been produced by individuals—a process known as “content analysis.”

In recent years, participation in online cancer communities by patients and their relatives (ie, posters) has become a subject of scientific investigation. We recently reviewed the impact of participation in online communities on patient-reported outcomes (questionnaires) [12]. However, as yet there has been no comprehensive overview of the quality of research into content analysis and subjects shared in cancer communities. Such an overview can be of great added value for patients, community service providers, health care providers, and researchers. They can learn from user-generated content, provided that their content is findable. We did not find any systematic review or study on this subject that has synthesized this information and identified trends across multiple online communities.

In this systematic review, we focus on content analysis of online cancer communities (group spaces) and not on blogs (personal spaces). The definition of content analysis as “a systematic, replicable technique for compressing many words of text into fewer content categories based on explicit rules of coding,” from Stemler’s paper [13], is an adequate starting point. Content analysis is a methodical means of gaining insight into several key aspects of user-generated content. For example, content analysis clarifies which kinds of information patients share with each other, as well as which characteristics of posters and linguistic aspects may influence the content.

### Objectives

The value of content analysis is that it enables people, for example, researchers and patients, to find relevant subjects in texts and to compare such texts with other texts over time. The content can be analyzed using qualitative, quantitative, or mixed methods [14,15]. Qualitative content analysis consists in methodically identifying themes and patterns in text by coding the content [16], whereas the essence of quantitative content analysis is counting words and recognizing patterns on the basis of the word counts, whereby involving context in the analysis, though sometimes difficult, is highly relevant [14,17]. By repeating content analysis in the same environment over a period of time, insight into possible trends can be gained.

In this systematic review, we address the following questions:

1. What is the quality of available research that analyzes user-generated content posted by cancer patients and their relatives?
2. If the quality of research is adequate, what kind of content do posters share with each other on the internet? For example, content of cancer, treatment, personal or emotional information.

## Methods

### User-Generated Content

For this systematic review, we have included peer-reviewed publications that describe content analysis of participation by posters in online cancer communities. In some cases, the online community is part of a broader online eHealth service, so that participants can also take part in other Web-based activities such as responding to questionnaires or participating in guided online support groups. An example of such a broader online eHealth service is the CHESS application (Center for Health Enhancement Systems Studies) with information, social support, and problem-solving tools [18]. The focus of content analysis is not the posters themselves but what they write: their posts, also referred to as “user-generated content” in online cancer communities. Evaluating other forms of Web-based contact (eg, blogs, chat sessions, Facebook posts, and Twitter tweets) is beyond the scope of this review.

### Search Strategy

We searched PubMed (MEDLINE), PsycINFO, the Cochrane Central Register of Controlled Trials, and ScienceDirect (the last search being in July 2017) on the following terms: (cancer patient) and (support group or health communities) and (online or internet), without any date parameters. PubMed automatically added the Medical Subject Headings terms (a hierarchically organized terminology for indexing and cataloging of biomedical information) with the synonyms of search terms necessary for a better selection of the PubMed literature. Subsequently, in July 2017, we tried to expand our results with the following additional terms: “online forum” or “message board” or “bulletin board.” We manually went through the first 100 most relevant results, which did not yield any new papers for this review.

To focus on the subject of our review, we decided that studies would be included according to all of the following criteria: (1) the publication was an original peer-reviewed paper (eg, no systematic reviews, book chapters, dissertations, poster abstracts, editorials, or letters to the editor); (2) it was written in English; and (3) the aim was content analysis of user-generated content of cancer communities. Studies were excluded if one of the following criteria applied: (1) they involved patient populations other than cancer patients and survivors; (2) they studied a structured online health intervention or the community was moderated by professionals; (3) they developed case studies, concepts, or models of content analysis, or (4) they studied patient-reported outcomes as a result of Web-based participation.

**Figure 1:**
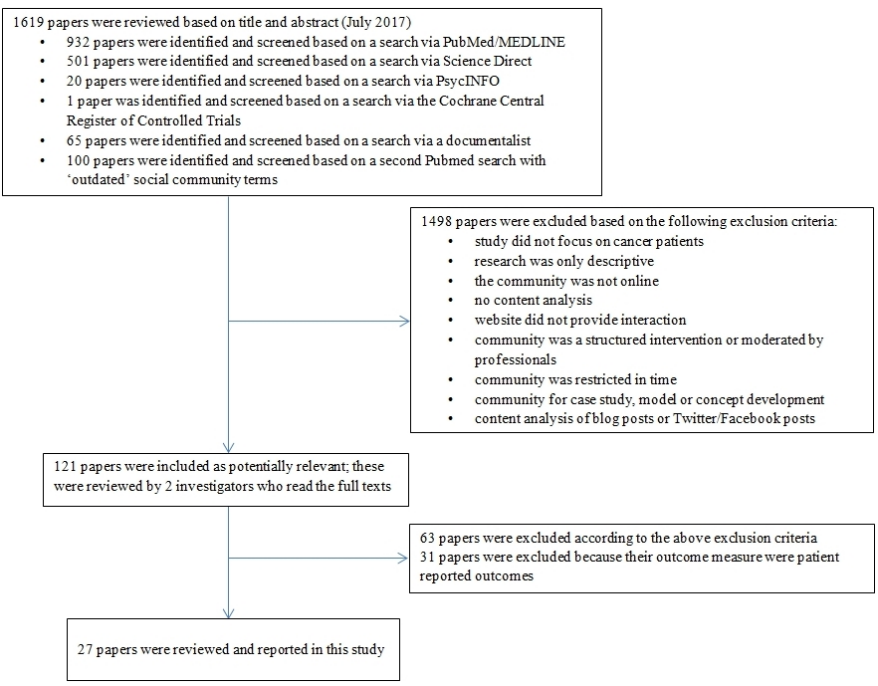
Flow chart of the literature search.

These inclusion and exclusion criteria were applied to our initial 1619 papers. After removal of duplicates and records not meeting the inclusion criteria, 121 records remained. Hard copies of these studies were obtained, and these were reviewed by 2 investigators (ME and FM) independently of each other. Both investigators checked the papers in detail on our predetermined inclusion and exclusion criteria. Each made their own decisions, and if they did not agree, they then discussed with each other in order to reach a final decision. Both reviewers also used citation tracking to identify other papers potentially eligible for inclusion. This did not yield any new records. The 2 investigators agreed with each other on the final selection of papers: 27 were found to be eligible for inclusion in this review. Figure 1 is a flowchart of this selection procedure.

### Quality Assessment

Both investigators (ME and FM) assessed the methodological quality of each of the selected studies using a 14-item standardized checklist based on established criteria for systematic review that are presented in Table 1 [19-21]. After reviewing 5 papers, we tailored the criteria list for reviewing papers related to content analysis in cancer communities. Each item of a selected paper that matched our criteria received either half a point or a full point, depending on its importance. This was to prevent items of lesser significance being too heavily weighted. If an item did not meet our criteria or was described insufficiently or not at all, 0 points were assigned. Item 14 would be probably difficult to satisfy for qualitative research papers.

The highest possible score was 9. The papers were then sorted into arbitrarily defined quality categories. Papers scoring 75% or more of the maximum attainable score (≥7.0 points) were considered to be of “high quality.” Studies scoring between 55% and 75% (5.0-6.5 points) were rated as being of “adequate quality.” Studies scoring equal to or lower than 55% (≤4.5 points) of the maximum attainable score were considered to be of “low quality.”

**Table 1:** List of criteria for assessing the methodological quality of studies.

Item no.	Item	Quality-points
1	Year of data collection is indicated	0.5
2	URL of website(s) or name of platform is indicated	0.5
3	Number of posts is indicated	0.5
4	Number of posters is indicated	0.5
5	A description is included of at least three variables of the community population (health/demographic)	0.5
6	A description is included of at least two community variables	0.5
7	Inclusion and/or exclusion criteria are described	1
8	Participation rates for patients are indicated and there are more than 50 posters	0.5
9	The study size is at least 1500 posts over 2 years (arbitrarily chosen)	0.5
10	The results of 2 or more groups are compared	1
11	The data collection process is described	1
12	The data analysis process is described	1
13	The data are described	0.5
14	Statistical proof for the main findings is reported	0.5
	Total	9

## Results

### Characteristics and Quality of the Included Studies

On the basis of our inclusion criteria, 27 studies were included in this review [22-48]. The quality scores ranged from 5.0 to 8.5 points, and the overall mean quality score was 6.8. The papers that present a combination of automated and manual analysis methods are of the highest quality (mean quality score=7.4; Table 2).

Of the 27 studies, 16 (59%) were found to be of high quality [24-29,31,37,39,41-44,46-48]. The remaining 11 studies (41%) were found to be of adequate quality [22,23,30,32-36,38,40,45] according to our criteria.

The studies were published between 1998 and 2016—most of them (15) in 2011 or later (Multimedia Appendix 1). The data collection occurred between 1996 and 2013 (Multimedia Appendix 1). Most of the studies (22) were conducted in the United States [23-29,31-37,39-41,43,44,46-48]. With 3 British studies [22,42,45] and 1 Australian [30], there were in total 26 from English-speaking countries (Multimedia Appendix 1).

In 17 studies [25-30,37,38,40,41], the researchers reported on which websites the analyzed content was found. Of those studies, 7 were part of the CHESS program [31,32,39,43,44,46,47]. In 2 cases, the researchers determined that the website URL could not be stated, for reasons of privacy [30,36].

Most studies described the number of posts ranging from about 200 [28] to 1.5 million [48] and the number of posters ranging from 75 [35] to 90,000 [48] (Multimedia Appendix 1). A total of 6 studies analyzed fewer than 1000 posts [22,26,28,33,35,41], and 6 studies analyzed more than 10,000 posts [31,32,40,42,47,48]. The studies analyzing large numbers of posts mainly relate to breast cancer, whereas those analyzing small numbers relate to other types of cancers.

Previous research [12] revealed that most of the active participants in online cancer communities are women, as proved to be the case in the studies included in this review. Among these studies, 11 examined the content only of a breast cancer community [27,31,32,39,41,43-48], 4 studies analyzed and compared posts of breast or prostate cancer communities [24,30,34,42], and one study compared posts about breast and intestinal cancer [40]. In 19 studies (70%), the posters’ ages were not given.

A recognized method of analysis is the systematic manual coding of content (ie, written text or spoken word) and retrieval of relevant topics on the basis of that coding in order to enable reporting [49]. Recent computer-based developments have made it possible to automatically analyze texts published on the internet.

**Table 2:** Mean score by analysis method.

Method of analysis	Mean quality score
All papers	6.8
Manual coding: [22-25,27-30,33-36,38,41,45]	6.6
Automated coding: [26,40,42-44,46,47]	6.9
Combination: [31,32,37,39,48]	7.4

In 15 studies, researchers coded the content only manually [22-25,27-30,33-36,38,41,45]. In 7 studies, researchers used only a computer tool for analysis [26,40,42-44,46,47], and in 5 studies a combination of manual and automatic analysis was used [31,32,37,39,48]. The CHESS authors mainly used Infotrend [31,32,39,46,47] and Linguistic Inquiry and Word Count (LIWC) [43,44], whereas the others used LIWC [26,48], WordSmith [42], and Sandalowski [35]. Portier reported using an algorithm that he devised himself [40]. Meier clearly indicated that he used annual thematic coding (according to ATLAS.ti [50]) but automatically determined the frequency of in-text occurrence [37]. This approach facilitates not only the processing of knowledge of context during coding but also its inclusion in the analyses. Wang et al showed that automatic coding and analysis of a large corpus (>1.5 million posts) is similar in quality to the manual coding of a small corpus, though the former yields more detailed information [48].

### Content Posters Share in Cancer Communities

After having listed the characteristics and quality of the included studies, we will now further investigate the findings of the studies.

Most of the authors used their own coding systems to analyze content (Multimedia Appendix 1). Therefore, there was no consistency in the employed codes, their categories, or the coding method. All the authors referred to 2 main categories: *informational* support and emotional support. Fifteen studies reported only on the content (ie, what the posters discussed) [23-25,27,30,31,33-36,38,42,43,45,46]. Six studies explicitly referred to social aspects (such as interaction between users) in addition to content [22,28,29,37,39,47]. Six studies focused on emotional changes, mainly as a result of posting and reacting to others’ posts [26,32,40,41,47,48]. In these cases, reply posts in reaction to previous posts within the same thread were found to produce an emotional change after some time—usually a positive change. Research by Mursch and Behnke-Mursch [38] showed that 15% of the posts discussed alternative treatment, an aspect that was not referred to in any of the other studies. For example, analysis revealed that fewer words of negative connotation tended to be used in later reply posts. A key question was whether it was reasonable to conclude from this decrease in negatively connoted words that the initial poster, following peer reaction to his or her original post, was feeling more positive.

To summarize, posters shared information on a wide variety of topics. In addition to informational support, often they also provided and obtained emotional support. Posters shared information, opinions, and experiences in relation to aspects, including their illness, the treatment, its side effects and other consequences, the quality of clinicians, alternative treatments, their emotions, and their relationships.

### Patient Characteristics

Some of the researchers combined results of content analysis with patient characteristics. Most of the studies that predicted content differences based on characteristics of posters were of high quality. In general, male patients tended to be more oriented toward informational support and female patients more toward emotional support [24,29,30,42]. In cases where posts by friends and family were separately analyzed on platforms, women tended to be more active than men. Friends and family were more oriented toward informational support than patients were [24,29,30], and in this context there was hardly any difference between men and women. When the patient had an unfavorable prognosis, posters were also more oriented toward informational support than when the patient had a favorable prognosis [25]. Posters who frequently used religious words in their posts had higher functional well-being scores [43]. Namkoong et al [39] found that there was a greater feeling of community “bonding” when people not only read but also wrote content. It should be noted that “being there for others” is extremely important for the sense of well-being. Wang et al [48] asserted that when there was emotional bonding, posters remained active in the community for longer than when there was only informational exchange. His research also showed that posters tended to request informational support directly and emotional support indirectly.

### Linguistic Approaches

Some high-quality papers also took a linguistic approach. Shaw et al [44] suggested that posters who more often used the personal pronoun “I” also tended to express negative emotions more often. Seale et al [42], who analyzed word use in offline qualitative interviews with 97 cancer patients (secondary use) and compared these with online posts, found that online posters used a broader vocabulary range than those interviewed offline. In addition, men used a greater variety of words when discussing medical matters, whereas women did so when discussing emotions. Regarding posts by young adults, Crook et al [26] showed that shorter sentences tended to yield more reactions. Use of the personal pronoun “I” yielded more reactions than the use of “we.” Verb tenses were also relevant: posts in future tense tended to have fewer reactions than posts in present or past tenses.

### Quality of User-Generated Content

In one study, the quality of user-generated content (correct/incorrect statements in the posts) was an important issue. Esquivel et al’s study [27] (of high quality) showed that incorrect advice was given relatively infrequently (10 of 4600 posts, ie, 0.22%) and was corrected quite soon after posting (ie, within 9 min to 9 hours). Esquivel et al can comment on the correctness of content because they had the posts coded by breast cancer experts. Sillence [45] gives another nuance: posters relatively infrequently (9%) made direct requests for advice. More frequently, there were requests for information or a personal opinion (34%), problems were disclosed (35%), or a question was formulated as a “same-boat” experience (20%).

### Limitations of Content Analysis

A practical limitation of automated content analysis is probably the “intelligence” of the tools. Counting letters and words is relatively trivial, but in order to generate context-relevant feedback, the tool must combine characteristics of the post and relate them to the coded label. This requires the use of machine learning methods, which must be devised and written by computer scientists in close collaboration with content experts for the different research areas. Portier et al [40] refer to an algorithm that they used (Multimedia Appendix 1), whereas Meier et al [37] clearly indicate that they have done the thematic coding manually (according to ATLAS.ti ) and have automated the process of determining the frequency of occurrence of certain terms in the text. Most of the papers on automated content analysis did not provide insight into topic lists or content themes. Only Wang et al [48] describe their working method in the greatest detail and offer developed knowledge, including their topic lists. An effectively functioning algorithm that can analyze contextually (in this case, knowledge about cancer and the healthcare system) would represent an enormous advance.

## Discussion

### Principal Findings

Patients and their relatives increasingly share experiences in online cancer communities, making this a very valuable resource not only for patients but also health care providers, researchers, and healthcare professionals. This paper made a systematic inventory of the kind of information that patients share online and of the methods used by researchers to analyze these user-generated content. We reviewed 27 studies, of which 15 studies were manually coded, 7 automated, and 5 used a combination of methods. The best results can be seen in the papers that used both analytical methods. All the authors referred to two main content categories: informational support and emotional support.

### Quality of Research

This review has shown that entirely automatic analysis of user-generated content of cancer posters is still relatively rare. Of the 27 authors [26,31,32,37,39,40,42-44,46-48], 12 analyzed content using an automated instrument of analysis, with or without manual coding. When they used such an automated instrument, they analyzed greater numbers of posts, often more than 10,000.

It is difficult to compare the various methods of analysis, and therefore, also their results. Researchers stated the names of the computer tools (see Multimedia Appendix 1 for methods of analysis and tools, [50-55]) they used but only briefly described how they worked. Some of the automated tools count words; others consider how far words are apart; still others use standardized wordlists and/or categories or make their own wordlists or themes (Multimedia Appendix 1, content themes). The researchers who coded manually mostly analyzed a smaller number of posts with more possibilities of contextual interpretation. The automated analysis gave information about patterns, changes in word use, and communication processes. This diversity of used methods of analysis—manual, automated, or a combination—and code themes (Multimedia Appendix 1) made it impossible for the reviewers to compare the results, let alone analyze how the type of tool affects the results obtained. We have found very few references to reviews of such tools [56].

### Qualitative Research With Professionals Enables Context

Automated analysis of content accuracy seems to us to be almost unfeasible without knowledge of the content. To determine the degree of accuracy, detailed knowledge of the subject area and correct interpretation of the posts is essential, and therefore, it requires that experts are involved in the process. Correct interpretation of the content is still very difficult for the automated analysis systems [14]. Esquivel et al [27] solve this problem by using 3 clinicians, including a breast cancer surgeon, to manually code the posts on accuracy. This methodological intervention enables research into a subject area about which there is much discussion in society. Understandably, some oncologists fear that patients may spread inaccuracies or falsehoods regarding their form of cancer online and thus unnecessarily alarm fellow sufferers. However, Esquivel et al study finds hardly any such inaccuracies or falsehoods [27], probably because people are generally “sensible” and do not request “advice” but information and experiences [45].

When professionals know that the accuracy of user-generated content is feasible, they can refer their patients to online communities. The information is not evidence-based, but it helps individual patients to empower [57] and can probably help to find some information about how others learned to cope with their rare problems [6,57]. There is a possibility for professionals to become a member of the community and share their knowledge. Another possibility is that professionals give answers on patients’ questions such as on kanker.nl [58]. This content is common knowledge of the total community.

### Future Opportunities

The results of this review reveal interesting opportunities, not only for relevant applications that can benefit patients and health care professionals [8] but also for academic researchers. Professionals can learn from patients’ narratives [59,60] and when professionals know that the accuracy of user-generated content is feasible, they can refer their patients to online communities [27]. To make user-generated content discoverable for cancer patients is a challenge. Search engines help patients find information, but the precision on the internet and within a website can be improved. For this, the algorithms have to be improved. User-generated content on the internet gives researchers access to experiences of patients, in a relatively simple way. They can provide insight into how patients deal with their illness over a longer period of time. The collection of data via questionnaires is often time intensive and has mostly a limited number of measurement moments [11].

Automated analysis also enables to compare validated medical information on the internet, with user-generated content on the same topic in discussion groups and blogs, or on Twitter and Facebook [61-63], and find omissions in medical information. Given the large amount of work involved in developing algorithms and their complexity, and in order to prevent knowledge and care institutions from becoming dependent on commercial companies, we think that more interdisciplinary collaboration within academia is highly recommended.

### Future Research of User-Generated Content

Content analysis of user-generated content in online communities is an emerging form of academic research. After all, it was not until about 20 years ago that (cancer) patients started sharing information about their illnesses online: for example, in 1995 via mailing lists of Association of Cancer Online Resources or Acor website (only cancer) [64] and in about 2005 in an entirely new way on PatientsLikeMe [65] (on all kinds of diseases). The balance between informational support and emotional support varies between the included studies, though the cause of such variance cannot be explained. To what extent this is due to the research methodology used and/or coding system and/or amount of posts or posters is also unclear. In-depth research is needed to draw conclusions on this matter. For example, we do not know whether content generated by a small community differs from content generated by a large community. Differences in activity of the community can also be understood in terms of different platform focuses. In addition, whether a community is moderated or not can also influence how and about what aspects the participants share.

### Limitations of This Study

A limitation of this review is that we compared both qualitative and quantitative research using the same checklist of quality criteria. Especially Q-criterion 14 (statistical proof for main findings reported) is arguably more applicable to quantitative than to qualitative research (although 6 out of 15 qualitative research method papers did in fact satisfy this criterion).

We did not include papers of other user-generated content types. The body of academic literature includes few publications on analysis of other types of user-generated content such as that of bloggers as well as Facebook and Twitter posters for cancer. They too share experiences that may be relevant to other patients and caregivers. Some of them have many followers, and therefore, also a relatively large impact. Also, sources such as blogs, Facebook, and Twitter can quite easily be incorporated by using automation.

### Conclusions

In conclusion, this review found that all included papers are of moderate (11 papers) or high (16 papers) quality. The papers with a combination of manual and automated content analysis are of the highest quality. With increasing number of cancer patients [9] who generate more content on the Internet, it is becoming increasingly important to make that knowledge of patients about their illnesses available to others. For the near future, the mixed method—combination of qualitative and quantitative analyzing methods—gives the best results. Maybe in the future, automated content analysis can be helpful to do this fast and also in an accurate manner.

The results of this review reveal interesting opportunities, not only for relevant applications that can benefit patients and healthcare professionals but also for academic researchers.

## Appendix

Multimedia Appendix 1 Characteristics of the peer-reviewed publications and quality score.

## Abbreviations

CHESS: Center for Health Enhancement Systems Studies
LIWC: Linguistic Inquiry and Word Count
