# Supplementary material for: Analysis of Content Shared in Online Cancer Communities: Systematic Review
Source: JMIR Cancer. 2018 Apr 3;4(1):e6. doi: 10.2196/cancer.7926 (PMC5904449; doi:10.2196/cancer.7926)
Supplement: Multimedia Appendix 1 [file cancer_v4i1e6_app1.pdf]

First author, year, Country	Years in which data collected	Posts (Posters)	Cancer	Method[a] and software tool[b]	Content themes used by coding	Conclusions	Q-score[c]
Algtewi [22] 2015 GB	2011	399	head & neck	A	informational support / esteem / network and emotional support / tangible assistance	The findings suggest that online support groups can be promising sources of head and neck cancer-related informational and emotional support for the recipients.	6.5
Beusterien [23] 2013 USA	2010-2011	1522 (264)	bowel	A (+ 2 [49])	side effects / treatment response and impact	Online colorectal cancer communities provide patients with convenient and valuable emotional support and disease information.	6
Blank [24] 2010 USA		3203	breast/ prostate	A	medical / intimacy / emotional support / economic / other	Findings identify several key differences in the use and benefits of online support sites for breast and prostate cancer.	7.5
Buis [25] 2011 USA	2005-2006	3717 (587)	several	A	informational support / emotional support	Relationships between survival rate and support types were significant. Results suggest that individuals experiencing low-survival-rate cancers may have a greater desire for informational support online than individuals experiencing high-survival-rate cancers.	8.5
Crook [26] 2016		684	several	B (3 [51])	not reported	Posts with replies contained fewer words per sentence, had more first-person pronouns, had more expressions of negative emotions, and contained more	7

USA						present tense and past tense verbs.	
Esquivel<br>[27]<br>2006<br>USA	2005	4600<br>(310<br>)	breast	A	not reported	Most posted information on breast cancer was accurate. Most false or misleading statements were rapidly corrected by participants in subsequent postings.	7
Gill<br>[28]<br>2012<br>USA	2012	218<br>(93)	ovary	A	not reported	Online discussion forums can play a crucial and indispensable role in dealing with diseases such as ovarian cancer, for which limited treatment options exist.	7
Ginossar<br>[29]<br>2008<br>USA	2000	1424	CLL/<br>Lung	A	information reply / information seeking / unsolicited information / emotional support / conflict / advocacy / other	Findings revealed (a) similarities in the content of communication in the two virtual communities, (b) gender differences in participation, and (c) differences in utilization of these online groups between patients and family members.	8.5
Gooden<br>[30]<br>2007<br>Australia	2004	1039<br>(164<br>)	breast/<br>prosta<br>te	A	informational support / emotional support	Both men's and women's dialogues fell into the categories of information support (women: 60%, men: 64%) and emotional support (women: 40%, men 36%).	5.5
Han<br>[31][d]<br>2011<br>USA	2001-<br>2003	1969<br>5<br>(231<br>)	breast	C (4<br>[52])	informational support / emotional support	Findings suggest that it is a combination of empathy expression and reception that is crucial to attaining optimal benefits for cancer patients. Empathic expression provides a salutary effect for patients who experienced a higher degree of concern associated with their cancer diagnosis and follow-up treatments.	8

Kim<br>[32][d]<br>2012<br>USA	?	19695<br>(177)	breast	C (4 [52])	physical and psychological / guilt / mortality / images of cancer / creating a positive attitude / healthcare / online social interaction / cancer survivorship	Supportive exchanges of receiving and giving play positive, but different, roles in predicting psychosocial health outcomes. Moreover, emotional support giving and receiving tend to reinforce each other.	5.5
Klemm<br>[33]<br>1998<br>USA	1996-1997	300<br>(97)	bowel	A	information giving/seeking / personal opinions / encouragement/support / personal experiences / thanks / humor / prayer / miscellaneous	The Internet Cancer Support Group is a means of offering support to cancer patients, their families and caretakers in a non-traditional format.	6.5
Klemm<br>[34]<br>1999<br>USA	1997	1541<br>(335)	prostate/<br>breast/<br>other	A	information giving or seeking / personal opinions / encouragement/support / personal experiences / thanks / humor / prayer / miscellaneous / activism	Attention should be given to facilitating the entry of underserved groups into Internet Cancer Support Groups.	5
Klemm<br>[35]<br>2008<br>USA	2006	300<br>(75)	several	A (+ 5)	not reported	Cancer survivors are utilizing Internet resources to find health-related information and support that has not been provided by healthcare providers.	5.5
Lobchuk<br>[36]<br>2015<br>USA	2008-2009	1406<br>(569)	lung	A	disease information / diagnostic test information / treatment information / symptoms / marked deterioration / advocacy / experiencing	The online support community is a valued, accessible avenue for information exchange and non-judgmental emotional support for individuals dealing with lung cancer.	6

					healthcare providers and the system / positive survivorship / making sense of emotions		
Meier [37] 2007 USA	2003-2004	2755 (1125)	several	C (6 [53])	specific treatment / communicating with health care providers / problem management strategies / coping with cancer recurrence	The most common expressions of support were offers of technical information and explicit advice about how to communicate with healthcare providers. Topics and proportions of informational and emotional support differed across the lists. This qualitative study shows that subscribers can and do find what they seek. They also find opportunities to play rewarding roles as support givers.	8.5
Mursch [38] 2003 Germany	2001-2002	3272 (380)	brain	A	not reported	The brain tumour mailing list is a communication medium for brain tumour patients and their care providers, which distributes and reproduces information of heterogeneous quality.	6
Namkoong [39][d] 2013 USA	2004-2006	5050 (236)	breast	C (4 [52])	not reported	This study shows that emotional support is more than something cancer patients receive; it is part of an active, complex process that can be facilitated by social media.	7
Portier [40] 2013 USA	2005-2010	29384	breast/ bowel	B (7 [54])	not reported	Using text-mining tools to assess sentiment, sentiment change and thread topics provides new insights that community managers can use to facilitate member interactions and enhance support outcomes.	5.5
Qiu	2009-2010	298	breast	A	not reported	This work establishes foundational concepts for further	7

[41]<br>2011<br>USA						studies of sentiment impact of Online Health Community (OHC) participation and provides insight useful for the design of new OHCs or enhancement of existing OHCs in providing better emotional support to their members.	
Seale<br>[42]<br>2006<br>GB	2001 + 2005	12000 (1150)	breast/ prostate	B (8 [55])	not reported	Web forums appear to be subjectively experienced by both sexes as relatively private places for the exchange of intimate personal information.	7
Shaw<br>[43][d]<br>2007<br>USA	2001-2003	(97)	breast	B (3 [51])	not reported	Several different religious coping methods were used and therefore being less afraid of death, finding blessings in their lives and appraising their cancer experience in a more constructive religious light.	7
Shaw<br>[44][d]<br>2008<br>USA	2001-2003	(97)	breast	B (3 [51])	not reported	A positive relationship was found between use of first-person pronouns and negative emotions.	7
Sillence<br>[45]<br>2013<br>GB	2011	1442	breast	A	not reported	The majority of the messages solicited advice or requests for information and opinion. A novel form of advice to "anyone in the same boat as me" was the use of personal experience.	6.5
Wang<br>[48]<br>2015<br>USA	2001-2011	1.562.459 (90.000)	breast	C (3 [51] / 1)	emotional self-disclosure (pos-neg) /<br>informational self-disclosure (pos-neg) / asking a question / eliciting support (emo	Self-disclosure is effective in eliciting emotional support, whereas question asking is effective in eliciting informational support. Moreover, perceptions that people desire	8

					- info support) / providing support (emo - info support)	particular kinds of support influence the support they receive. Finally, the type of support people receive affects the likelihood of their staying in or leaving the group. These results demonstrate the utility of machine learning methods for investigating the dynamics of social support exchange in online support communities.	
Yoo [46][d] 2013 USA	2005-2007	5065 (192)	breast	B (4 [52])	not reported	Expressing emotional support changed in a quadratic trajectory, with a range of factors predicting the changing pattern of expression.	7.5
Yoo [47][d] 2014 USA	2005-2007	18064 (236)	breast	B (4 [52])	not reported	Giving and receiving emotional support in computer-mediated social support groups has positive effects on emotional well-being for breast cancer patients with higher emotional communication, while the same exchanges have detrimental impacts on emotional well-being for those with lower emotional communication competence.	7

[a] A = manual B = automated C = combination of manual and automated

[b]1= Latent Dirichlet Allocation (NA), 2= Maxqda, 3= LIWC [50], 4= Infotrend, 5= Sandalowski by Waltz (NA), 6= Atlas.ti, 7= Ada boost, 8= WordSmith

[c] Quality-score [d] CHESS-study

References Appendix

22. Algtewi EE, Owens J, Baker SR. Analysing people with head and neck cancers’ use of online support groups. Journal of Psychosocial Research on Cyberspace. 2015;9 (4)(article 1).

23. Beusterien K, Tsay S, Gholizadeh S, Su Y. Real-world experience with colorectal cancer chemotherapies: patient web forum analysis. Ecancermedicalscience. 2013;7:361. PMID: 24143155. doi: 10.3332/ecancer.2013.361.

24. Blank TO, Schmidt SD, Vangsness SA, Monteiro AK, Santagata PV. Differences among breast and prostate cancer online support groups. Computers in Human Behaviour. 2010;26 (2010) 1400-4.
25. Buis LR, Whitten P. Comparison of social support content within online communities for high- and low-survival-rate cancers. Computers, informatics, nursing : CIN. 2011 Aug;29(8):461-7. PMID: 21876394. doi: 10.1097/NCN.0b013e318214093b.
26. Crook B, Glowacki EM, Love B, Jones BL, Macpherson CF, Johnson RH. Hanging by a thread: exploring the features of nonresponse in an online young adult cancer survivorship support community. Journal of cancer survivorship : research and practice. 2016 Feb;10(1):185-93. PMID: 26123806. doi: 10.1007/s11764-015-0465-8.
27. Esquivel A, Meric-Bernstam F, Bernstam EV. Accuracy and self correction of information received from an internet breast cancer list: content analysis. BMJ 2006 22 april;332 939-42.
28. Gill PS, Whisnant B. A qualitative assessment of an online support community for ovarian cancer patients. Patient related outcome measures. 2012;3:51-8. PMID: 23185122. doi: 10.2147/PROM.S36034.
29. Ginossar T. Online participation: A content analysis of differences in utilization of two online cancer communities by men and women, patients and family members. Health communication. 2008;23(1):1-12.
30. Gooden RJ, Winefield HR. Breast and prostate cancer online discussion boards: a thematic analysis of gender differences and similarities. J Health Psychol. 2007 Jan;12(1):103-14. PMID: 17158844. doi: 10.1177/1359105307071744.
31. Han JY, Shah DV, Kim E, Namkoong K, Lee SY, Moon TJ, Cleland R, Bu QL, McTavish FM, Gustafson DH. Empathic exchanges in online cancer support groups: distinguishing message expression and reception effects. Health communication. 2011 Mar;26(2):185-97. PMID: 21318917. doi: 10.1080/10410236.2010.544283.
32. Kim E, Han JY, Moon TJ, Shaw B, Shah DV, McTavish FM, Gustafson DH. The process and effect of supportive message expression and reception in online breast cancer support groups. Psycho-oncology. 2012 May;21(5):531-40. PMID: 21416553. doi: 10.1002/pon.1942.
33. Klemm P, Reppert K, Visich L. A nontraditional cancer support group. The Internet. Computers in nursing. 1998 Jan-Feb;16(1):31-6. PMID: 9454992.
34. Klemm P, Hurst M, Dearholt SL, Trone SR. Gender differences on Internet cancer support groups. Computers in nursing. 1999 Mar-Apr;17(2):65-72. PMID: 10194883.
35. Klemm P. Late effects of treatment for long-term cancer survivors: qualitative analysis of an online support group. Computers, informatics, nursing : CIN. 2008 Jan-Feb;26(1):49-58. PMID: 18091621. doi: 10.1097/01.NCN.0000304753.41493.f4.
36. Lobchuk M, McClement S, Rigney M, Copeland A, Bayrampour H. A Qualitative Analysis of "Naturalistic" Conversations in a Peer-Led Online Support Community for Lung Cancer. Cancer nursing. 2015 Sep-Oct;38(5):E21-31. PMID: 25254411. doi: 10.1097/NCC.0000000000000207.
37. Meier A, Lyons EJ, Frydman G, Forlenza M, Rimer BK. How cancer survivors provide support on cancer-related Internet mailing lists. Journal of medical Internet research. 2007;9(2):e12. PMID: 17513283.
38. Mursch K, Behnke-Mursch J. Internet-based interaction among brain tumour patients. Analysis of a medical mailing list. Zentralblatt fur Neurochirurgie. 2003;64(2):71-5. PMID: 12838475. doi: 10.1055/s-2003-40375.
39. Namkoong K, McLaughlin B, Yoo W, Hull SJ, Shah DV, Kim SC, Moon TJ, Johnson CN, Hawkins RP, McTavish FM, Gustafson DH. The effects of expression: how providing emotional support online improves cancer patients' coping strategies. Journal of the National Cancer Institute Monographs. 2013 Dec;2013(47):169-74. PMID: 24395987. doi: 10.1093/jncimonographs/lgt033.
40. Portier K, Greer GE, Rokach L, Ofek N, Wang Y, Biyani P, Yu M, Banerjee S, Zhao K, Mitra P, Yen J. Understanding topics and sentiment in an online cancer survivor community. Journal of the National Cancer Institute Monographs. 2013 Dec;2013(47):195-8. PMID: 24395991. doi: 10.1093/jncimonographs/lgt025.
41. Qiu B, Zhao K, Mitra P, Wu D, Caragea C, Yen J, Greer GE, Portier K. Get Online Support, Feel Better. Sentiment Analysis and Dynamics in an Online Cancer Survivor Community. IEEE; San Francisco, California. Privacy, Security, Risk and Trust (Passat)2011.
42. Seale C, Ziebland S, Charteris-Black J. Gender, cancer experience and internet use: a comparative keyword analysis of interviews and online cancer support groups. Social science & medicine (1982). 2006 May;62(10):2577-90. PMID: 16361029. doi: 10.1016/j.socscimed.2005.11.016.
43. Shaw B, Han JY, Kim E, Gustafson D, Hawkins R, Cleary J, McTavish F, Pingree S, Eliason P, Lumpkins C. Effects of prayer and religious expression within computer support groups on women with breast cancer. Psycho-oncology. 2007 Jul;16(7):676-87. PMID: 17131348. doi: 10.1002/pon.1129.
44. Shaw BR, Jeong Yeob H, Hawkins RP, McTavish FM, Gustafson DH. Communicating about self and others within an online support group for women with breast cancer and subsequent outcomes. J Health Psychol. 2008 Oct;13(7):930-9. PMID: 18809644. doi: 10.1177/1359105308095067.

45. Sillence E. Giving and receiving peer advice in an online breast cancer support group. Cyberpsychology, behavior and social networking. 2013 Jun;16(6):480-5. PMID: 23659724. doi: 10.1089/cyber.2013.1512.
46. Yoo W, Chih MY, Kwon MW, Yang J, Cho E, McLaughlin B, Namkoong K, Shah DV, Gustafson DH. Predictors of the change in the expression of emotional support within an online breast cancer support group: a longitudinal study. Patient education and counseling. 2013 Jan;90(1):88-95. PMID: 23122429. doi: 10.1016/j.pec.2012.10.001.
47. Yoo W, Namkoong K, Choi M, Shah DV, Tsang S, Hong Y, Aguilar M, Gustafson DH. Giving and Receiving Emotional Support Online: Communication Competence as a Moderator of Psychosocial Benefits for Women with Breast Cancer. Computers in human behavior. 2014 Jan;30:13-22. PMID: 24058261. doi: 10.1016/j.chb.2013.07.024.
48. Wang YC, Kraut RE, Levine JM. Eliciting and receiving online support: using computer-aided content analysis to examine the dynamics of online social support. Journal of medical Internet research. 2015 Apr 20;17(4):e99. PMID: 25896033. doi: 10.2196/jmir.3558.
49. Maxqda, The art of data analysis. https://www.maxqda.com/: VERBI GmbH; 2018 [cited 2018 02-25]; Available from: http://www.webcitation.org/6xUgsB115
50. Pennebaker JW, Francis ME, Booth RJ. Linguistic inquiry and word count: LIWC 2001. : Mahway: Lawrence Erlbaum Associates; 2001.
51. LIWC2015, the golden standard in computerized text analysis. http://liwc.wpengine.com/: Pennebaker Conglomerates, Inc. ; 2018 [cited 2018 02-25]; Available from: http://www.webcitation.org/6xUh214c6.
52. InfoTrend!, Providing Solutions, Creating Trends. http://infotrendusa.com/: Infotrend Inc (ITI); 2018 [cited 2018 02-25]; Available from: http://www.webcitation.org/6xUhH4L2G.
53. ATLAS.ti, Qualitative Data Analysis. http://atlasti.com/: ATLAS.ti Scientific Software Development GmbH; 2018 [cited 2018 02-25]; Available from: http://www.webcitation.org/6xUhVeUAm.
54. AdaBoost, a predictive algorithm for classification and regression. https://nl.mathworks.com/discovery/adaboost.html?requestedDomain=www.mathworks.com: The MathWorks, Inc.; 2018 [cited 2018 02-25]; Available from: http://www.webcitation.org/6xUhh4WGR.
55. WordSmith Tools, Windows software for finding word patterns. http://www.lexically.net/wordsmith/: Lexical Analysis Software Ltd; 2018 [cited 2018 02-25]; Available from: http://www.webcitation.org/6xUhouLBl.
